# It Takes a Village: How Community-Based Peer Support for Breastfeeding Bolsters Lactation Prevalence Among Black Mississippians on the Gulf Coast

**DOI:** 10.3390/pediatric16040091

**Published:** 2024-11-23

**Authors:** John P. Bartkowski, Katherine Klee, Xiaohe Xu, Jacinda B. Roach, Shakeizia “Kezi” Jones

**Affiliations:** 1 Department of Sociology and Demography, University of Texas at San Antonio, San Antonio, TX 78249, USA; xiaohe.xu@utsa.edu; 2 Bartkowski & Associates Research Team, San Antonio, TX 78258, USA; kklee182@gmail.com; 3 Mississippi Public Health Institute, Madison, MS 39110, USA; jroach@msphi.org (J.B.R.); kjones@msphi.org (S.K.J.)

**Keywords:** breastfeeding, lactation, race, ethnicity, peer, support, disparities, rural, social, perceptions, southern, Mississippi

## Abstract

Background/Objectives: Breastfeeding rates are considerably lower among African American women and across the U.S. South. Our study introduces the concept of *community-based peer support for breastfeeding*, as measured through beliefs about women’s comfort breastfeeding in various social situations (i.e., in the presence of women and men as well as close friends and strangers). Methods: We examine if community-based peer support for breastfeeding is associated with reported lactation prevalence in primary social networks among survey respondents living on the Mississippi Gulf Coast. Special attention is paid to racial differences in the breastfeeding support–prevalence relationship. We use data drawn from a survey that combines a random sample of adults who are representative of the Mississippi Gulf Coast population and a non-random oversample of African Americans in this predominantly rural tri-county area. Results: Analyses of data from wave 1 of the CDC-funded 2019 Mississippi REACH Social Climate Survey reveal low overall levels of African American breastfeeding network prevalence (knowing friends and family who have breastfed). However, community-based peer support for breastfeeding significantly amplifies breastfeeding network prevalence for black Mississippians when compared with their white counterparts. Discussion: Previous research has indicated that breastfeeding promotional messages have a limited impact on African American breastfeeding propensity along the Mississippi Gulf Coast. However, the current study indicates that enhanced community-based peer support for breastfeeding can be a key facilitator for improved lactation outcomes among African Americans as compared with whites. Conclusion: We establish that breastfeeding is best conceived as both an interpersonal encounter (an activity often conducted in the presence of others) and a collective achievement (a practice influenced by community norms). We discuss study implications and directions for future research.

## 1. Introduction

Breastfeeding and human milk consumption are highly recommended for infant feeding and nutrition. The American Academy of Pediatrics and World Health Organization recommend exclusive breastfeeding during the first six months after birth, with complementary foods introduced at six months of age [1]. In the United States, 1 in 12 infants (8.6% of live births) were categorized as low birthweight in 2022, with an average of 86 Mississippi infants born at low birthweight per week [2]. Mississippi also has the highest annual preterm birth rate at 14.8%—i.e., 4.4% higher than the national average—and is one of only eight U.S. states with a preterm birth rate of over 11.5% [3]. March of Dimes has most recently provided Mississippi with a report card grade of F given the high preterm birth rate and infant mortality rate (9.4/1000 live births; 330 infant deaths in 2021) [3]. The Mississippi rate of preterm birth is highest for Black infants (17.7%), followed closely by American Indian/Alaska Natives (13.9%), Whites (12.6%), Hispanics (11.5%), and Asian/Pacific Islanders (10.2%) [4]. Mississippi also has a breastfeeding initiation rate of 63.2% as of 2018–2019, with 69.4% of infants having been breastfed for any duration [5,6]. However, Mississippi falls below the U.S. national average (84.1%) in breastfeeding initiation by more than 20%, with a 34% disparity between racial/ethnic groups compared to the 16.7% national disparity [7]. The largest national and Mississippi disparities are primarily among Asian and Black or African American mothers and infants. Non-Hispanic Black women exhibit some of the lowest breastfeeding rates among all ethnic groups in the United States [7,8]. Mothers of preterm infants are more likely to initiate lactation compared with mothers of term infants but are also more likely not to meet their intended breastfeeding goals due to a variety of barriers (e.g., delayed lactogenesis, reduced milk production, competing demands on their time, and NICU visitation) [9,10,11,12].

### 1.1. Benefits and Barriers Associated with Breastfeeding

Breastfeeding is a protective factor for a variety of infectious, atopic, and cardiovascular diseases in addition to leukemia, necrotizing enterocolitis, celiac disease, and inflammatory bowel disease. Breastfeeding reduces the development of type 1 and 2 diabetes, obesity, hypertension, and cardiovascular disease, all of which are high health risk indicators among Mississippi mothers [13,14]. Among low-income communities, benefits often span reduced infant diarrhea, respiratory infection mitigation, and low mortality. Breastfeeding has also been established to positively impact neurodevelopment, reducing the risk of mental health disorders [15,16,17,18]. There are further health benefits of breast milk consumption related to specific bioactive components, such as leptin which has been correlated to infant protection from excess weight gain, among other health concerns [19].

Studies have shown several barriers to breastfeeding across racial groups but especially among non-Hispanic African American women. African American mothers are often knowledgeable about the infant benefits of breastfeeding, where convenience and benefits for infants are primary reasons for breastfeeding. However, fear of difficulty and pain are among the main breastfeeding deterrents that African American women cite, especially in low-income environments [8]. In response to the general decline in breastfeeding related to different factors including the production of manufactured breastmilk substitutes, the World Health Organization introduced the 1980 WHO International Code of Marketing of Breastmilk Substitutes [20]. The 1990 Innocenti Declaration on Breastfeeding followed and was subsequently renewed in 2005 to redouble efforts for breastfeeding awareness and promotion [21]. However, commercial milk formula marketing continues to saturate pregnancy-related healthcare experiences and divert pathways to breastfeeding [22]. While social media marketing for breastfeeding is prevalent among African American mothers in Mississippi, there is a decrease in breastfeeding experiences compared to their white peers [23]. Furthermore, studies on women’s experience with public breastfeeding often included extensive challenges, such as creating a spectacle of breastfeeding, breast sexualization, bystander discomfort, and efforts to breastfeed in hidden areas [24]. Barriers such as C-section occurrence, postoperative pain, lactation issues, and pregnancy complications compounded mothers’ social and demographic barriers and lack of professional support. The presence of short maternity leave, no job-related or professional support for breastfeeding, and food insecurity significantly, often negatively, influence breastfeeding initiation and duration [25]. Alternatively, breastfeeding experience-enhancing themes encompass having a supportive audience and confidence in breastfeeding [26]. Increasing women’s self-efficacy is attributed to successes in African American mothers meeting breastfeeding goals, as well as addressing implicit bias in healthcare providers and maternal care nurses throughout the prenatal and postpartum breastfeeding process [27,28].

### 1.2. Social Determinants of Breastfeeding

Breastfeeding-related social determinants are outlined at the individual, interpersonal, community, organizational, and policy levels. At the individual level, breastfeeding is noted as a valued behavior often hindered by exhaustion, isolation, and time constraints. At an interpersonal level, social media, peer-to-peer, and family relations act as breastfeeding support network opportunities. At the community level, in one study, participants identified cultural acceptance of breastfeeding as a potential support and barrier [29]. Organizationally, hospitals may have supportive breastfeeding-friendly policies in place but lack breastfeeding-knowledgeable personnel. However, there is a greater call for breastfeeding legislation to establish and enforce protections for workplace breastfeeding [29]. WIC, healthcare organizations, and local community agency interventions result in the highest likelihood of improved breastfeeding outcomes among African American mothers [30]. Additionally, healthcare staff lactation education increases maternal/infant-trained staff knowledge of breastfeeding and the application of maternal support roles. Staff education encourages a change in attitude toward the use of human milk among preterm infants [30,31]. Such facilitators are essential as NICU teams play critical roles in ongoing lactation support by providing education, institutional support, and medical support. Similarly, workplace lactation education programs are also effective in supporting breastfeeding duration among employed mothers and partners of employed fathers [31]. Fathers of infants play a principal role in breastfeeding support and promotion. One study noted that fathers’ perspectives on breastfeeding support are often shaped by their knowledge of breastfeeding and participation in the process of breastfeeding [32,33]. Research indicates that verbal encouragement from loved ones to new mothers increases breastfeeding duration and exclusivity, especially among non-white mothers.

### 1.3. Community-Based Peer Support for Breastfeeding

Similar to loved ones’ support, peer-to-peer support is crucial to the success of breastfeeding. In one study, the Hispanic Health Council created a Breastfeeding Heritage and Pride program to address community barriers to breastfeeding, including lack of relatable role models, limited social support, public embarrassment, lack of breastfeeding knowledge, and local community encouragement, with generally positive effects [34]. Education and counseling are most successfully delivered by peer counselors, generally defined as women who have successfully breastfed, have similar cultural roots and life experiences as the currently breastfeeding mothers, and have completed training on lactation management and communication skills. Community peer support may also include International Board-Certified Lactation Consultants who provide clinical guidance and ongoing training to peer counselors, as well as direct support to clients. Peer support is also a known facilitator for reducing social isolation [35]. In the age of virtual communities, mothers are seeking breastfeeding information through a variety of online pathways. In one rural U.S. study, participants were assessed using telelactation or in-person services for breastfeeding-related peer support and health consultations. A majority of rural participants in the telelactation group (56%) reported exclusive breastfeeding at 12 weeks, whereas 45% of participants in the control group (in-person) reported exclusive breastfeeding at 12 weeks [36]. A pilot study for mobile breastfeeding support revealed that women valued experiencing an easy-to-navigate, interactive design that was reassuring and included evidence-based, credible sources of breastfeeding information [37]. Mothers who are breastfeeding often rely on virtual connections when they experience feelings of isolation, lack of professional support, or prefer online support more than face-to-face contact. Online support results in mother empowerment and changes in breastfeeding outcomes and perception, similar to effects observed for those who participate in face-to-face or in-person support [38,39,40]. Members of a private Facebook group noted a distinct increase in self-efficacy regarding how the group shared in women’s experiences and aided mothers in their breastfeeding journeys. Further facilitation of greater breastfeeding duration and success includes education, accessibility, online community support, normalization, and extended goals [40,41]. Additionally, communities offering remote breastfeeding support reduce by 25% the risk of women ending exclusive breastfeeding at 3 months [42].

Mississippi has seen a burgeoning presence of breastfeeding-positive initiatives. Between 2014 and 2020, Mississippi hospitals designated as Baby-Friendly increased from 0 to 22, simultaneously increasing breastfeeding initiation from 56% to 66% through the Mississippi CHAMPS (Communities and Hospitals Advancing Maternity Practices) initiative [43]. Additionally, Delta Healthy Sprouts based in rural Mississippi produced generally successful but mixed results [44]. Their findings indicated a significant increase in breastfeeding knowledge scores from baseline to the late gestational period, but higher breastfeeding belief scores only for those who initiated breastfeeding compared to those who did not. The Delta Healthy Sprouts intervention revealed the drawbacks of increasing knowledge and addressing modifiable barriers, such as social norms and lack of social support, without adopting support modifications. Mississippi ranks last in most health outcomes across the United States and is often compared with some of the world’s most impoverished countries [3,4,5,6,7]. Mississippi’s health climate sets a crucial backdrop for this study with potentially replicable results and paths forward for global areas with similar health outcomes. Mississippi also provides a stringent test case for promising approaches that could transform breastfeeding and related health outcomes in locales of concentrated social disadvantage. Our study uniquely examines African American, other non-white, and white racial differences related to (1) perceived community-based peer support for breastfeeding and (2) breastfeeding social network prevalence. Our findings expand current knowledge of the complex relationship between race, breastfeeding support networks, and community contexts of breastfeeding in Mississippi.

## 2. Materials and Methods

The current study analyzes valid data that were previously used in another published article on breastfeeding media messaging differences by race [24]. The present study, however, has a very distinct focus compared to that previous publication, which did not explore the racial implications of community-based peer support for breastfeeding and lactation network prevalence. A cross-sectional survey design was used in that previous study to predict exposure to breastfeeding promotional information and reported prevalence of breastfeeding in primary social networks. This design was chosen because the Mississippi REACH Social Climate Survey is a baseline assessment of an overall project that continued through 2023. This snapshot was intended to elucidate the contours and antecedents of African-American/white disparities in breastfeeding along the Mississippi Gulf Coast. Data collection approval was received and governed by an existing memorandum of understanding between the Declaration of Helsinki and the Institutional Review Board of Mississippi State University (IRB-17-04-MOU).

In this study, we reconceive breastfeeding community support by grounding this concept in interpersonal relationships and practical experiences. We recognize that community support for breastfeeding is sometimes generalized, as is the case with the CDC Project First Consumer Opinion Panel survey: “In general, how supportive of breastfeeding are people in your community?” Response options for this item range from very supportive to very unsupportive. This question has strong face validity but relies on a nebulous definition of support that we aim to overcome. Therefore, we intentionally adopt a different approach to perceived community support for breastfeeding in this study. Breastfeeding is first and foremost a social practice (routine behavior), one that involves interpersonal relationships between mother, baby, and others within their social circle, including close women friends, close men and women friends, coworkers, acquaintances, and even strangers. Consequently, we contend that perceived community support is better conceptualized by gauging survey respondents’ beliefs about *how comfortable women in the community are breastfeeding in specific circumstances*. Ideally, these circumstances would account for both proximate (close) and distal (not so close) social relationships.

Consequently, our study uses a different set of questions originally introduced on the CDC Project First Consumer Opinion Panel to examine community support for breastfeeding through perceptions of women’s comfort with breastfeeding in the presence of various peer groups. Our measure recognizes breastfeeding as an interpersonal encounter embedded in a cascading series of social relationships that range from close friends to strangers in public settings. This redefinition provides a more stringent test of *actual community support* because it prioritizes *breastfeeding as a socially embedded practice*, which we surmise would be directly related to women’s comfort breastfeeding in a range of situations (close women friends, close men and women friends, and men and women acquaintances or strangers). A community could be seen as supportive of breastfeeding in principle based on the conventional operationalization of community support. However, that “support” means little if the actual practice of breastfeeding would create discomfort for mothers in these specific circumstances. Our more grounded and empirically precise redefinition of community support accounts for peer-based interpersonal affirmation or stigmatization of breastfeeding as might be experienced by mothers engaged in this practice. Our situationally focused definition of breastfeeding support is drawn from a CDC survey of validated items, but we also cross-checked face validity (question stem and response option comprehensibility) by securing feedback from select representatives of our population before fielding the survey.

### 2.1. Data Collection and Sample

Data for this analysis came from a cross-sectional survey, titled the Mississippi REACH Social Climate Survey. This survey was designed to gauge nutrition, tobacco use, infant feeding, and breastfeeding knowledge, attitudes, community support for breastfeeding, and lactation network prevalence among adults residing in the Mississippi Gulf Coast region. Participants in this study completed the Mississippi REACH Social Climate Survey created and managed by the Mississippi Public Health Institute [45,46]. The Mississippi REACH Social Climate Survey, through the REACH grant received by MSPHI, was funded by the Centers for Disease Control and Prevention’s Racial and Ethnic Approaches to Community Health (CDC REACH) program. The REACH program supported the implementation of the Healthy Families, Mothers, and Babies Initiative [47,48]. The Mississippi REACH Social Climate Survey examined racial disparities for residents in Hancock, Harrison, and Jackson counties along the Mississippi Gulf Coast. This area was prioritized for its high prevalence of poverty and other adverse social determinants of health. Health risks in these areas are further amplified for African American residents. African American representation in these counties is varied at 8.4% (Hancock), 26.2% (Harrison), and 21.6% (Jackson). County poverty rates are also high in the priority counties of Hancock (15.0%), Harrison (19.3%), and Jackson (15.7%), where the African American poverty rate in Mississippi overall is consistently 2.5 times that of whites [49].

The random digit dialing method using cell phone numbers was employed to draw a representative sample of adults aged 18 and above from three Mississippi Gulf Coast counties, namely Hancock, Harrison, and Jackson. This random sample was complemented by an oversample of African American males and females aged 18–50. A total of 80,000 relevant population numbers were chosen from a pool of 1,083,000. A maximum of eight dialing attempts were employed per telephone number before retirement. A total of 419 adults completed the survey with a cooperation rate of 38.7% and 7.9%, respectively, for the general adult population and the oversampled African American adult population. The survey was fielded from July through September 2019. Survey administration and data collection were approved by the Institutional Review Board of Mississippi State University (IRB-17-04-MOU).

### 2.2. Variables

The dependent variable is breastfeeding network prevalence indicated by a single survey item. Respondents were asked the following: “How common is breastfeeding infants among your female friends and relatives?” Response categories were recoded into (0) none of them have breastfed, (1) very few of them have breastfed, (2) about one-quarter of them have breastfed, (3) about half of them have breastfed, (4) a majority of them have breastfed, and (5) all of them have breastfed. Other responses were recoded as missing values. This measure has been used effectively in previously published research [24].

The focal independent variable is self-reported racial/ethnic identity. All responses were recoded into two dummy variables for (1) African Americans and (2) other racial/ethnic groups with whites as the reference. The moderating variable is perceived community-based peer support for breastfeeding, which was indicated by three survey items. Respondents were asked the following: “How comfortable do you believe women in your community are in the following situations?” (1) nursing a baby in the presence of close women friends, (2) nursing a baby in the presence of men and women who are close friends, and (3) nursing a baby in the presence of men and women who are not close friends. All responses were recorded on a Likert scale with 1 = very uncomfortable, 2 = somewhat uncomfortable, 3 = neither uncomfortable nor comfortable, 4 = somewhat comfortable, and 5 = very comfortable. These three items, taken from the CDC Project First Consumer Opinion Panel, were combined into a mean-score index with higher scores representing greater levels of perceived community-based peer support for breastfeeding. As explained above, we contend that this redefinition of community-based peer support through women’s comfort breastfeeding in the presence of various peer groups is a superior means of measuring this important construct. The Cronbach’s Alpha coefficient for this index was 0.683. Statistical controls included age (ranging from 18 to 83), gender (dummy-coded with males as the reference), annual household income (ranging from 1 = less than USD 10,000 to 11 = USD 200,000 or more), educational attainment (ranging from 1 = never attended school or only attended kindergarten to 9 = beyond master’s degree), and employment status (dummy-coded with other as the reference).

### 2.3. Analytical Approach

The survey data were weighted to represent adults 18 years of age and older residing in the Gulf Coast region of Mississippi (Jackson, Hancock, and Harrison counties) in 2019. The data were also weighted to adjust for over-sampled African American males and females aged 18–50. The multiple imputation method was used to estimate and replace all missing values in the dependent, independent, and control variables after confirming they were missing completely at random (MCAR) except for household income (used as a statistical control).

The dependent variable—breastfeeding network prevalence—was utilized roughly as a continuous variable. As such, we estimated three nested Ordinary Least Squares (OLS) regression models to assess how community-based peer support affects breastfeeding network prevalence, net of race/ethnicity and other statistical controls, and how community-based peer support for breastfeeding intersects with race/ethnicity in moderating African Americans’ low rate of breastfeeding network prevalence. As shown below, Model 1 was estimated to demonstrate a lower breastfeeding network prevalence for African Americans compared with whites, net of statistical controls. Model 2 established a positive association between community-based peer support for breastfeeding and lactation network prevalence after race/ethnicity and other statistical controls were included in the model. Finally, in Model 3, we added two interaction terms to show how community-based peer support for breastfeeding could amplify lactation network prevalence for African Americans as well as for other racial/ethnic groups when compared with whites after controlling for sociodemographic characteristics. Unless stated otherwise, all statistical analyses were conducted using the Statistical Package for the Social Sciences (SPSS) version 29 [50].

## 3. Results

Table 1 reports descriptive statistics for the Mississippi REACH Social Climate Survey participants. As can be seen from the table, 48.9% and 42.2% of the respondents are white and African American, respectively, whereas only 8.8% self-identified as other races. Since African American respondents were oversampled in this study, the proportion of African American respondents is still higher than that of the 2020 census (37.9%, including black alone or in combination) even after the data were weighted. Slightly more than one half of the respondents are female (50.6%) and 63% were employed at the time of the survey. On average, respondents are 43 years of age, have an educational background at some college or vocational school level, and have an average annual household income of more than USD 35,000 in 2018. In terms of breastfeeding network prevalence, the average value of 2.56 indicates that about one-quarter to one-half of friends in their primary networks have breastfed. Concerning the level of community-based peer support for breastfeeding, the reported average value is 3.28, which is in between the categories of neither unsupportive nor supportive and somewhat supportive.

**Figure 1:**
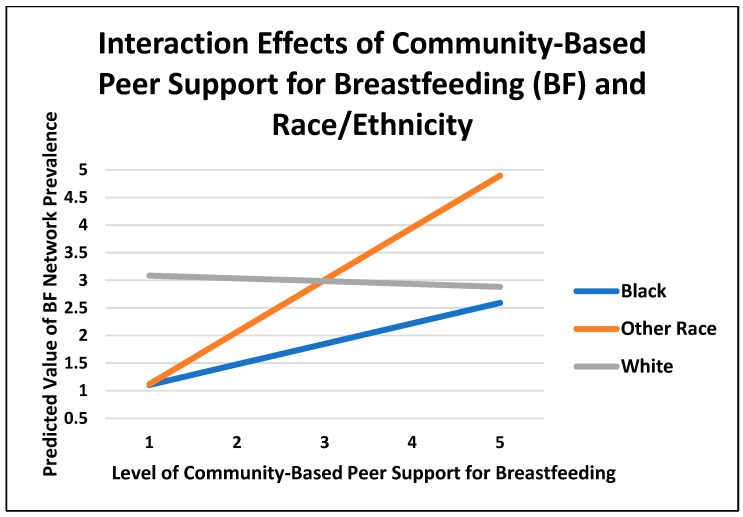
Interaction effects of community-based peer support for breastfeeding and race/ethnicity.

Table 2 displays the OLS regression results. As shown in Model 1, the regression coefficient for African American respondents indicates that on average, breastfeeding network prevalence is 0.978 units lower (*p* < 0.001) for African Americans than for whites, net of statistical controls. This statistically significant result is consistent with previous research findings that reveal a lower rate of breastfeeding networks for African Americans than for whites. Model 2 features the independent and positive effect of perceived community-based peer support for breastfeeding on lactation (breastfeeding) network prevalence. That is, for a one-unit increase in community-based peer support for breastfeeding, the expected lactation network prevalence increases by a factor of 0.204 regardless of racial/ethnic identity and other sociodemographic characteristics. Model 3 reports the interaction effects involving race/ethnicity and community-based peer support for breastfeeding. For African American respondents, the lower level (compared to whites) of lactation network prevalence is improved by a factor of 0.422 (*p* < 0.05), whereas for other racial/ethnic groups, the level of lactation network prevalence is further enhanced by a factor of 0.995 (*p* < 0.05), net of statistical controls. To visualize these interaction effects, Figure 1 shows that although African Americans reported a lower level of lactation network prevalence, lactation network prevalence increases as community-based peer support for breastfeeding increases relative to whites. It is interesting to observe that such an increase is more dramatic for other races (e.g., a steeper regression slope) when compared with whites. For whites, by contrast, lactation network prevalence declines somewhat as the level of community-based peer support for breastfeeding increases. Taken together, Table 2 and Figure 1 demonstrate that the positive effect of community-based peer support for breastfeeding on lactation network prevalence is amplified for African American and other racial/ethnic survey respondents as compared with whites. These results underscore the importance of community-based peer support for breastfeeding for racial/ethnic minority groups in Mississippi.

## 4. Discussion

Breastfeeding is a nuanced social interaction between mother and infant, healthcare workers, loved ones, and the broader community. That broader community includes proximate relationships (close women and men friends) as well as distal contacts (acquaintances and strangers). While international protocols have been established to limit the impact of formula on social messaging, formula is still a formidable barrier against breastfeeding [20,21]. Such messaging leads to a lack of breastfeeding engagement among healthcare workers, families, and those in other social networks [22]. Community-engaged programs, especially those that surround women with lactation-affirming peers, make a concerted effort to change the breastfeeding narrative by offering mothers readily available, credible resources of lactation experts and breastfeeding peers [34,35,36]. Our study results indicate that breastfeeding/lactation network prevalence (friends, family, and acquaintances who have breastfed) remains low among African Americans compared to their white counterparts. However, as community-based peer support for breastfeeding increases, so too does breastfeeding network prevalence increase across African American and other non-white racial groups. In contrast, community-based peer support for breastfeeding—measured here through perceptions of women in the community’s comfort breastfeeding in various social situations—is correlated with a minor decrease in lactation network prevalence among whites. We have contended that our more grounded and experiential definition of community-based peer support for breastfeeding is superior to a generic operationalization of this construct (“In general, how supportive are people in your community of breastfeeding?”). How could the impact of this seemingly minor divide in community support on network prevalence be explained? What difference might specific forms of community-based peer support offer?

First, community-based peer support and engagement are often formed to address a specific population or primary audience. The largest at-risk population in Mississippi is African American mothers, followed by Native American, Hispanic, and Asian American populations [3,7,8]. Mississippi’s Racial and Ethnic Approaches to Community Health’s (MS REACH) first round of funding from 2018 to 2023 prioritized addressing health disparities (especially breastfeeding) evident among African American women of childbearing age but served all populations along the designated coastal region. Community partnerships, outreach activities (e.g., community baby showers), and lactation support groups included African American women with breastfeeding experience. The results of this study are aligned with previous research which indicates community outreach strategies are effective for increasing breastfeeding network support and prevalence among African Americans and other non-whites [30,31,34].

Second, Mississippi is a predominately rural state. Rural environments often lack many opportunities for social network expansion and healthcare access without devoted community efforts [36,44]. Further, African American mothers are less likely than their white counterparts to have social networks (e.g., friends, family, and healthcare workers) with a high prevalence of breastfeeding [27,28,30]. Establishing peer support groups, online forums, and other community activities creates a space for African American and other non-white mothers to build social networks around breastfeeding [34,35,36,37,38,39,40,41,42]. Consistent with results from previous studies, our findings indicate that strengthening the broader community is integral to building African American social networks that support mothers throughout their breastfeeding journey, from first inquiry to varied periods of duration [30,31,32]. Addressing the breastfeeding support gap with peer group solutions in Mississippi communities is crucial and building networks for isolated or first-generation breastfeeding African American mothers may be especially efficacious. Community-based peer support networks offer a unique opportunity for a high-health-risk population of women to experience breastfeeding support in otherwise formula-focused healthcare and primary social network settings. These peer-based solutions, of course, could be effectively complemented by pro-breastfeeding messages, online forums, and access to professional lactation consultants. Community-based support avenues of various types are not mutually exclusive, though peer networks might be the most critical element among them.

Finally, MS REACH is one of several racial minority-focused breastfeeding initiatives in Mississippi [43,44,51,52,53]. All programs have representation of non-white mothers on their websites and in their activities. Research has pinpointed that utilizing relatable leaders, spokespersons, and healthcare workers makes the topic of breastfeeding more approachable and actionable for African American (and other non-white) mothers [30,31,35,43]. It is theoretically possible that the opposite can also occur. As such, not viewing or interacting with relatable figures in the community could limit breastfeeding dialogue in white social networks. Including primarily non-white racial/ethnic figures in campaigns, media messaging, and other aspects of community outreach may increase lactation prevalence among non-white groups but perhaps not white social networks. This increase in non-white breastfeeding support representation may result in a decrease in white representatives, which may then lead to a decrease in breastfeeding promotion and information-sharing among white networks. However, this possibility is only speculative and requires further investigation. It is possible that breastfeeding influences operate in ways that are distinct across racial/ethnic groups given structural disparities in breastfeeding supports. It is imperative to note that the slight decrease in reported white social network breastfeeding prevalence is not wholly reflected in the actual breastfeeding among white Mississippians, where whites have significantly higher percentages of initiation than African American or Asian infants [3,4,5,6,7]. A previous publication from the MS REACH Social Climate Survey Wave 1 revealed that whites were exposed to considerably less media messaging for breastfeeding but reported a higher prevalence of breastfeeding in their social networks [24]. By contrast, African Americans experienced increased media messaging exposure to breastfeeding promotion but reported lower breastfeeding prevalence. Therefore, African Americans remain a priority population to address breastfeeding health disparities, especially compared with their white counterparts. Our findings underscore the benefits of community-based peer support for breastfeeding among non-whites, especially African Americans, in encouraging breastfeeding information-sharing and prevalence.

There are a few limitations to our study, all of which are pathways for future research. This study analyzes data from a baseline assessment and provides a momentary snapshot of Mississippi Gulf Coast breastfeeding network disparities between whites and African Americans. However, our methodology, resources, and labor power did not include the ability to thoroughly test survey items for content validity considerations for our specific survey group (Gulf Coast Mississippians). We therefore relied on face validity assessments prior to survey administration and note that our breastfeeding community support items are drawn from a CDC survey with rigorously established construct validity. Future research would benefit from expanding on our findings by including item validity tests for specific populations. We also invite further comparisons of generalized versus situationally specific forms of community breastfeeding support, as we have argued strongly here for the benefits of the latter (operationalized through degrees of social comfort breastfeeding in the presence of various others). Additionally, we included breastfeeding media messaging avenues (newspapers, flyers, social media, doctor’s offices, etc.) in the overall analysis of the Social Climate Survey. However, we did not analyze racial/ethnic-specific breastfeeding campaign strategies (i.e., white and non-white representatives, cultural talking points). Therefore, it is difficult to know for certain that non-white community-based peer support is adversely affecting white breastfeeding network support. In future studies, integrating qualitative methods into data collection would benefit additional studies examining this facet of breastfeeding. Utilizing individual and focus group interviews related to Mississippi peer support by mothers with lived experience would refine key strategies in community breastfeeding engagement. Finally, this survey was conducted before the COVID-19 pandemic and formula shortages. A second wave of the Social Climate Survey was conducted in 2023 to assess how the COVID-19 pandemic and formula shortage impacted breastfeeding knowledge, dialogue, and experiences. Therefore, a future study utilizing this second wave of data to assess post-pandemic breastfeeding would be essential to analyzing more forward-looking practices in the field.

## 5. Conclusions

Our study examined racial differences in community-based peer support for breastfeeding and lactation network prevalence in Mississippi. We reconceived of community support for breastfeeding by stressing the peer advantage in lactation support, namely people’s beliefs about women in the community feeling comfortable breastfeeding in various social situations (diverse peer groups). We contend that this more grounded definition actually measures collective affirmation of breastfeeding as a normatively welcome and desirable practice. The relationship between community-based peer support for breastfeeding and lactation network support is overall positive for non-white racial/ethnic groups and (most notably for our purposes) African Americans. However, as community-based peer support for breastfeeding increases, lactation network support decreases among whites. This slight decrease in white social network support does not negatively impact the overall breastfeeding prevalence in Mississippi, which is among the lowest in the United States [5,6,7]. African Americans, followed closely by those in other non-white racial and ethnic groups, remain at high risk for mother and infant health disparities that would otherwise be reduced by breastfeeding. Therefore, a persistent emphasis on African American and other non-white breastfeeding promotion is needed. Additional to this study’s empirical findings, our results highlight the success of innovative methodological approaches, including the use of an African American oversample, a peer-focused definition of community support for breastfeeding, and a lactation network prevalence indicator that can be suitably answered by both men and women survey respondents. Future research would benefit by pursuing replicable results among populations with low breastfeeding prevalence and high infant health risk factors (i.e., low birthweight) outside of Mississippi. Until then, perceptions of women’s comfort breastfeeding in the presence of various social groups, both proximate and distal, are one promising avenue for bolstering lactation prevalence in communities that exhibit low breastfeeding rates.

## Figures and Tables

**Table 1:** Sample characteristics.

	Weighted *n*	Percent	Mean	SD
Lactation (BF) Network Prevalence			2.56	1.66
African American	177	42.2		
Other Race	37	8.8		
White	205	48.9		
CB Peer Support for BF (index)				
Age				
Male	207	49.4	3.28	0.97
Female	212	50.6	43.00	16.89
Household Income in 2018				
Education				
Employed	264	63.0	5.93	2.71
Not Working	155	37.0	4.95	1.46

Note: CB = Community-Based; BF = Breastfeeding.

**Table 2:** OLS regression models to predict lactation network prevalence (weighted).

Variable	Model 1 ^1^		Model 2		Model 3	
African American (reference: white)	−0.978	***	data	***	data	***
Other Race (reference: white)	0.167		0.194		0.303	
CB Peer Support for BF (index)			0.204	*	-0.051	
CB Peer Support for BF x AA					0.422	*
CB Peer Support for BF x Other Race					0.005	*
Age	0.003		0.003		0.001	
Female (reference: male)	0.198		0.217		0.260	
Household Income in 2018	0.018		0.020		0.025	
Education	0.106		0.092		0.105	
Employed (reference: other)	0.435	*	0.426	*	0.407	*
Intercept	1.848	***	1.229	*	2.040	***
*F*	9.259	***	9.830	***	9.070	***
*R* ^2^	0.136		0.161		0.182	
Weighted *n*	419		419		419	

^1^ * *p* < 0.05, ** *p* < 0.01, *** *p* < 0.001. Note: CB = Community-Based; BF = Breastfeeding.

## Data Availability

Data available on request due to restrictions (e.g., privacy or ethical). The data are not publicly available due to the data being held in a private repository.
